# Experimental Quantum Chemistry: A Hammett‐inspired Fingerprinting of Substituent Effects

**DOI:** 10.1002/cphc.202001053

**Published:** 2021-02-22

**Authors:** Francesco Sessa, Martina Olsson, Fredrik Söderberg, Fang Wang, Martin Rahm

**Affiliations:** ^1^ Department of Chemistry and Chemical Engineering Chalmers University of Technology SE-412 96 Gothenburg Sweden; ^2^ Department of Chemistry University of Rhode Island 140 Flagg Road Kingston Rhode Island 02881 USA

**Keywords:** chemical bonding, chemical descriptors, energy decomposition analysis, linear free energy relationships, reactivity prediction

## Abstract

The quantum mechanically calculable *Q* descriptor is shown to be a potent quantifier of chemical reactivity in complex molecules – it shows a strong correlation to experimentally derived field effects in non‐aromatic substrates and Hammett σ_m_ and σ_p_ parameters. Models for predicting substituent effects from *Q* are presented and applied, including on the elusive pentazolyl substituent. The presented approach enables fast computational estimation of substituent effects, and, in extension, medium‐throughput screening of molecules and compound design. An experimental dataset is suggested as a candidate benchmark for aiding the general development and comparison of electronic structure analyses. It is here used to evaluate the experimental quantum chemistry (EQC) framework for chemical bonding analysis in larger molecules.

## Graphical abstract

**The quantum mechanically calculable Q descriptor** is shown to be a potent quantifier of chemical reactivity in complex molecules – it shows a strong correlation to experimentally derived Field and Hammett σ parameters. The method enables for fast computational estimation of substituent effects and may prove useful for screening and design of compounds. An experimental dataset is suggested as a common benchmark for the development of electronic structure analyses.

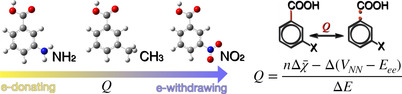

## 1 Introduction

In this work, we explore a quantum chemically derived descriptor for quantifying the electronic effects of functional groups. Knowledge of electronic and steric effects of functional groups, condensed in the form of descriptors, has been important ever since the advent of modern chemistry.[^1^, ^2^, ^3^, ^4^] Ideally, well‐chosen chemical descriptors allow us to both rationalize and predict the chemical properties of compounds and the outcome of reactions, thus aiding the development of functional molecules and synthetic procedures. Successful applications of descriptors abound, for example, in the design of pharmaceuticals and materials,^[5]^ for predicting trends in reaction rates[^6^, ^7^, ^8^] and for gaining insight into reaction mechanisms.^[9]^ Our approach, which focuses on the analysis of substituents on aromatic and non‐aromatic substrates, is both inspired by and is here first validated against the well‐known Hammett σ scale.^[10]^

The Hammett σ constant is an empirical chemical descriptor defined by the linear free energy relationship known as the Hammett equation [Eq. (1)]:^[10]^

(1) $$\log K=\log K_{0}+\rho \sigma ,$$

where *K* and *K_0_* are reaction equilibrium constants for a substituted and an unsubstituted benzoic acid derivative, respectively, σ is a constant that depends on the nature of the substituent and ρ is a constant that depends on the reaction mechanism and environment. Equation (2) expresses the corresponding Hammett relationship for rate constants, k and $k_{0}$ .

(2) $$\log k=\log k_{0}+\rho \sigma ,$$

Since its conception more than 80 years ago, the Hammett equation has provided chemical insight into the connection between electronic structure and reactivity. The presumption behind the Hammett equation is that the contribution of a given substituent, quantified by σ, shows little dependence on the reaction conditions and mechanism, as long as the nature of the substituent remains unchanged by such conditions. For example, the σ value of a substituent does not change between substituted benzoic acids, esters, and amides.^[10]^ Using this simple empirical approximation, Hammett was able to provide reliable quantitative estimates of electronic substituent effects.[^3^, ^10^] Today, the Hammett scale constitutes an important experimental basis for useful chemical concepts such as charge transfer, which cannot always be uniquely defined in quantum mechanics.^[11]^ As such, the Hammett scale is also a valuable experimental testing ground for theoretical descriptors meant to quantify different aspects of electronic structure.

Hammett parameters have been predicted using theory and can also be used as experimental benchmarks to valid descriptors produced by theory. One approach for evaluating σ from first principles is to calculate the Δ*G* of benzoic acid dissociation to obtain p*K*_a_ values.[^12^, ^13^, ^14^] However, because of the intricacies of modeling solvation effects accurately, computational prediction of p*K*_a_ is both challenging and costly.^[12]^ It is often faster, and sometimes more informative, to instead predict σ parameters by connecting them to other properties of the electronic structure. An early example is a work by Jaffé,^[15]^ where σ of substituents were shown to correlate with the electron density at the *meta* and *para* carbon atoms in the phenyl ring. Several studies have estimated σ from atomic charges obtained from quantum mechanical calculations on different benzene derivatives.^[16]^ DiLabio *et al*.^[17]^ demonstrated a relationship between the molecular ionization potential of disubstituted benzenes and the σ values of their substituents. Politzer and co‐workers have shown that the local average ionization energy can correlate with Hammett σ constants.[^8^, ^18^] Gadre *et al*.^[19]^ and Galabov *et al*.^[20]^ followed a somewhat similar approach, where local electrostatic potentials were compared to σ. Takahata *et al*.[^14^, ^21^] have shown how σ is related to the shift of carbon core levels due to the presence of a substituent. Liu *et al*. have proposed a generalized equation to connect the Hammett equation with Pauling's electronegativity.^[22]^ Popelier *et al*. have combined topological analysis of the electron density with principal component analysis to build a partial least square regression model able to estimate σ.^[23]^ Fernández and Frenking have pointed out a correlation between π‐conjugation energy and σ in substituted benzyl anions.^[6]^ Krygowski and co‐workers have worked to develop a physical interpretation of substituent effects through the use of Energy Decomposition Analysis (EDA) and by comparing σ with various quantities obtainable by quantum mechanical calculations.^[24]^ Before introducing the *Q* descriptor, which we study in this work, and its relationship to reactivity, we will briefly review its underlying theoretical framework.

### 1.1 Experimental Quantum Chemistry and a Different Take on the Chemical Bond

The descriptor that we will study is defined within an EDA scheme based on the following equation:[^25^, ^26^]

(3) $$\Delta E=n\Delta \bar{\chi }+{\Delta V}_{NN}-{\Delta E}_{ee},$$

where ΔE is the change in total energy over a chemical or physical transformation,n is the number of electrons and $\Delta \bar{\chi }$ is the change of the average electron energy. Previous work has shown that the $\bar{\chi }$ term of Eq. (3) can be attributed to the central chemical concept of electronegativity^[27]^ and $\Delta \bar{\chi }$ to electronegativity equalization.^[28]^ The $\Delta \bar{\chi }$ term can interchangeably, and approximately, be interpreted as the average change in the occupied molecular orbital energy over a transformation. The Δ*V_NN_* and Δ*E_ee_* terms in Eq. (3) quantify changes to electrostatic repulsion between equally charged particles, nuclei, and electrons, respectively. The latter two terms will both increase in magnitude when atoms are brought together. However, because Eq. (3) contains the negative of the *ΔE_ee_* term, the value of the combined electrostatic terms, Δ(*V*_NN_‐*E*_ee_), mostly cancels out during bond formation or cleavage. The non‐zero value of the Δ(*V*_NN_‐*E*_ee_) term can often be attributable to changes in the electronic distribution occurring because of chemical bonding. The reason why the multielectron term *ΔE_ee_* shows up with a negative sign in Eq. (3) is related to the double‐counting of this energy in the $\Delta \bar{\chi }$ term.^[25]^

The terms of Eq. (3) can be calculated at any quantum chemical level of theory, but they can also, in principle, be *estimated*, directly or indirectly, from a combination of thermochemistry, photoelectron, and vibrational spectroscopies and diffraction experiments.[^25^, ^26^] Whereas Eq. (3) is exact within the Born‐Oppenheimer approximation, the ability to estimate its component terms from experimental data merits the label of “Experimental Quantum Chemistry”, or EQC. A more detailed description of EQC is provided in Ref. [25] and Ref. [26].

In the EQC framework a chemical bond can be defined by its homolytic bond dissociation reaction. In such a definition, the energy associated with a chemical bond directly relates to the corresponding energy change over the transformation, Δ*E*. This definition of a chemical bond and its energy is not necessarily the most elegant or practical.^[29]^ For example, it is not always possible to consider a homolytic bond formation or dissociation of an intramolecular bond. One could also imagine defining a chemical bond based on its heterolytic dissociation. However, such a choice makes apparent the issue of reference states – how to choose the “right” cationic and anionic fragments that are formed. There exist many other EDA methods (see, e. g., Refs.[^25^, ^30^, ^31^, ^32^] and references therein), which are capable of defining interaction energies between parts of a molecule and to partition these energies into interpretable contributions such as, for example, electrostatic, charge transfer and dispersion interactions.[^30^, ^33^] Defining a chemical bond by its homolytic formation/dissociation, like we do, has one advantage: such a process is, at least in principle, possible to observe experimentally. In what follows, we will rely solely on Density Functional Theory (DFT) calculations for our chemical bonding analysis.

### 1.2 The Bond Descriptor *Q*

One way to summarize information from the EQC‐EDA shown as Eq. (3) is through a descriptor we denote as *Q*:^[26]^

(4) $$Q\;=\left(n\Delta \bar{\chi }-\Delta \left(V_{NN}-E_{ee}\right)\right)/\Delta E.$$

*Q* is designed to weigh the orbital $n\Delta \bar{\chi }$ and electrostatic Δ(*V*_NN_‐*E*_ee_) contributions to the total energy and can be considered an index capable of distinguishing between different chemical transformations. In particular, *Q* has shown an ability to differentiate between covalent, polar, and ionic bonds in diatomic molecules, which points to a connection with charge transfer.^[26]^ The magnitude of *Q* for homolytic bond formation/dissociation has also been shown to correlate strongly with the degree of correlation energy in diatomic bonds. We point the reader to Ref. [26] for a more detailed discussion on the interpretation of *Q* for diatomic bond formation.

*Q* has been shown to correlate to physical observables also in larger molecules.[^28^, ^34^] Sessler *et al*. have, for example, studied the formation of intra‐ and intermolecular hydrogen bonds in α,α‐difluorotoluene derivatives and showed that *Q* can distinguish red‐ and blue‐shifted hydrogen bonds.^[28]^ Fugel *et al*. have used *Q* to investigate the homolytic formation of X−O bonds in H_n_XOH compounds, where X is any second or third‐row element.^[34]^ Also, in these larger molecules, *Q* is able to classify X−O bonds in what is arguably a chemically meaningful manner. However, the physical interpretation of *Q* becomes less straightforward in larger molecules.

In this work, we take the next step in the application of EQC to large molecules and use *Q* to probe the character of chemical bonds inside of substituted benzoic acids and substituted bicyclo[2.2.2]octane carboxylic acids. In order to see how *Q* relates to electron‐donating and withdrawing effects of substituents, we compare with experimental data in the form of Hammett σ constants, as well as field (*F*) and resonance (*R*) effects of functional groups.^[3]^

## 2 Results and Discussion

**Figure 1:**
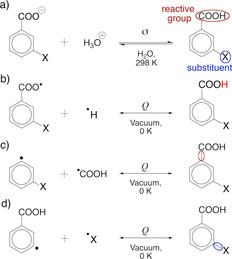
a) The standard measure of Hammett *σ* is the change in the p*K*_a_ of benzoic acids in aqueous solution relative to a reference where the substituent is hydrogen, viz. Eq. (1). *Q* is independent on the direction of a reaction (viz. Eq. 4) and is in this work calculated for the homolytic bond dissociation/formation of: b) the carboxylic O−H bond, c) the Ar−CO_2_H bond, and d) the aryl‐substituent bond.

In Figure 1a, we show the most common reaction used for determining σ values, the acid‐base equilibrium of benzoic acids in water. In what follows, we will investigate substituted benzoic acids in a related manner by comparing calculated *Q* values for bonds in these molecules with experimental σ constants. One critical question we ask is which bond in a substituted benzoic acid should be analyzed to obtain the most relevant information on a molecule's reactivity?

Panels b, c, and d of Figure 1 illustrate the different kinds of bonds in benzoic acids investigated in this work, and these bonds will be separately described in the following three sections. Towards the end of this work, we additionally address non‐conjugated systems and discuss how *Q* relates to two main components of substituent effects, field and resonance. The *Q* descriptor does not appear well suited to describe the creation of charged species, and we, for this reason, do not consider any heterolytic dissociations that would be more akin to the acid‐base equilibrium shown in Figure 1a.

### 2.1 The Nature of the Carboxylic O−H Bond

**Figure 2:**
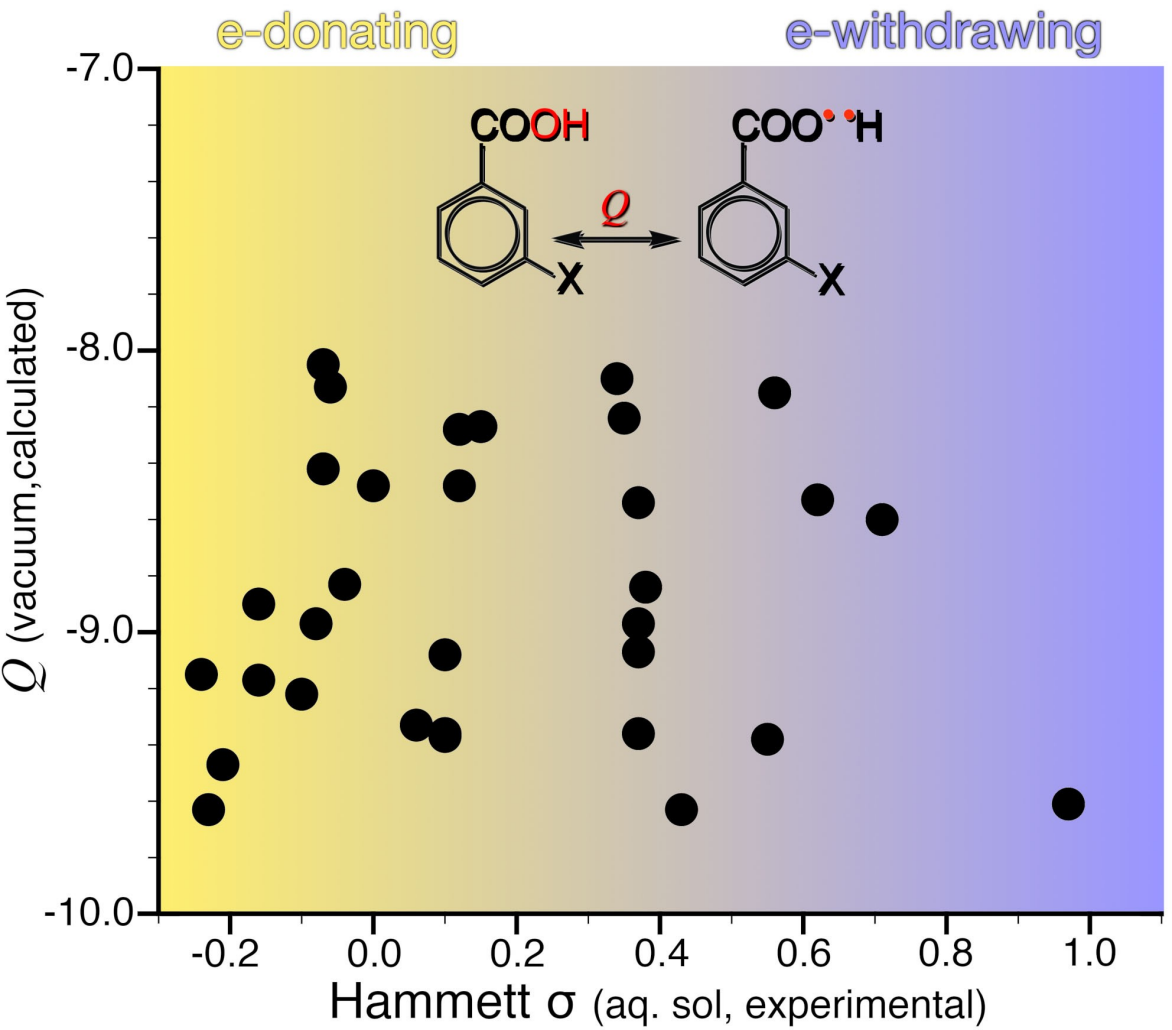
The *Q* descriptor calculated for the indicated carboxylic O−H bond plotted against the Hammett *σ* constant of meta‐substituted benzoic acids shows no correlation.

The carboxylic O−H bond defines the most acidic site of the molecule – it is the bond that is heterolytically broken upon proton transfer – and we, therefore, expect the nature of this bond to relate to acidity. We probe the nature of this bond by calculating *Q* for its homolytic dissociation, as shown in Figure 1b. We know from previous work that the value of *Q* is sensitive to charge transfer in the homolytic formation of diatomics,^[26]^ and so our first results shown in Figure 2 are surprising: there is *no* correlation between *Q* calculated for the O−H bond and σ! Of course, we should keep in mind that *Q* and σ are far from the same quantities. The Hammett σ constant is an empirical estimate of the effect of a substituent on the acidity of benzoic acids at 298 K in aqueous media. In contrast, *Q* here refers to a bond formation/dissociation process, occurring in vacuum as T→0 K, without consideration of thermal or solvent effects. Nevertheless, even as *Q* is a *process*‐related descriptor, we do expect it to provide information on bond properties, such as polarity.^[26]^ So, what is going on?

The O−H bond differs from the bonds shown as Figures 1c and 1d in several respects: the O−H bond is broken during deprotonation, and it is also not part of the π‐conjugated system. A substituent affects acidity primarily by changing the balance in stability between the benzoic acid and its conjugate base. Information on the reactivity of benzoic acids might therefore be better obtained by studying the dissociation of intramolecular bonds that are common to both the acid and its conjugated base. Examples of such bonds are those between the aromatic moiety and the reactive carboxyl group or the substituent functional group, shown in Figures 1c and 1d, respectively.

### 2.2 Connecting the Carboxyl Bond to Benzoic Acid Reactivity

**Figure 3:**
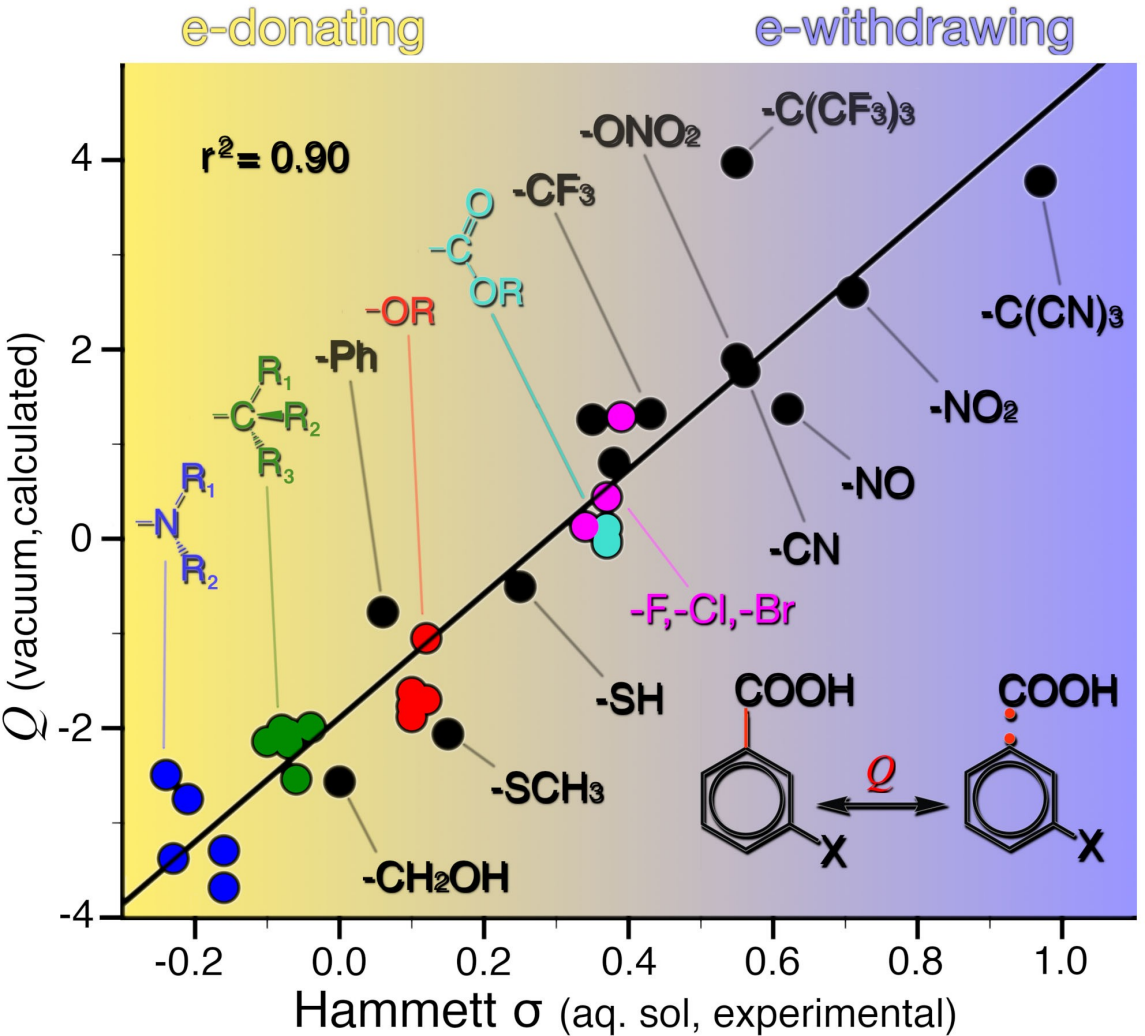
The *Q* descriptor calculated for the indicated carboxyl bond plotted against the Hammett *σ* constant of meta‐substituted benzoic acids. Colored circles indicate clustering of related chemical groups.

We next look at *Q* calculated for the bond to the carboxyl group. In other words, homolytic dissociations of the general type shown in Figure 1c. Figure 3 highligths a strong (r^2^=0.90) correlation between σ and *Q*, when the latter is calculated for 35 benzoic acids with common substituents in meta position.^[3]^ We attribute the one distinct outlier of Figure 3, the C(CF_3_)_3_ group, to the group's bulkiness, as it slightly distorts the geometry of the phenyl ring by bending the *ortho*‐hydrogens. That no significant steric effects are present is a necessary condition for the Hammett equation to be applicable. A corresponding plot for *para*‐substitutions shows qualitatively the same trend, albeit with a slightly different coefficent of determination (r^2^=0.83, Figure S1).

Should we be surprised by the striking correlation of Figure 3? Yes and no. On the one hand, we know that the value of *Q* is related to charge transfer.^[26]^ On the other hand, and as mentioned, *Q* and σ are fundamentally different quantities. Even considering that σ is assumed independent of temperature and solvent,^[10]^ the degree to which *Q* and *σ* correlate in Figure 3 is noteworthy.

In Figure 3, colored circles represent substituents’ families with a similar chemical function. Both alkyl and ether groups (green and red, respectively) produce tight clusters of data points. For substituent groups that feature amino or carbonyl/carboxyl functions (blue and cyan, respectively) the results are slightly more spread on the *Q* axis. The clustering of *Q* values for similar compounds correctly predicts a small difference in chemical reactivity within each family of substituents. Nevertheless, the spread in the *Q* scale for similar groups is larger than what is seen in the σ‐scale. One reason for the scattering is arguably subtle differences within a family of substituents, for example, the length of alkyl chains. We will return to discuss other reasons why groups with similar σ can feature different *Q* values.

### 2.3 The Sensitivity of *Q* to Local Effects

**Figure 4:**
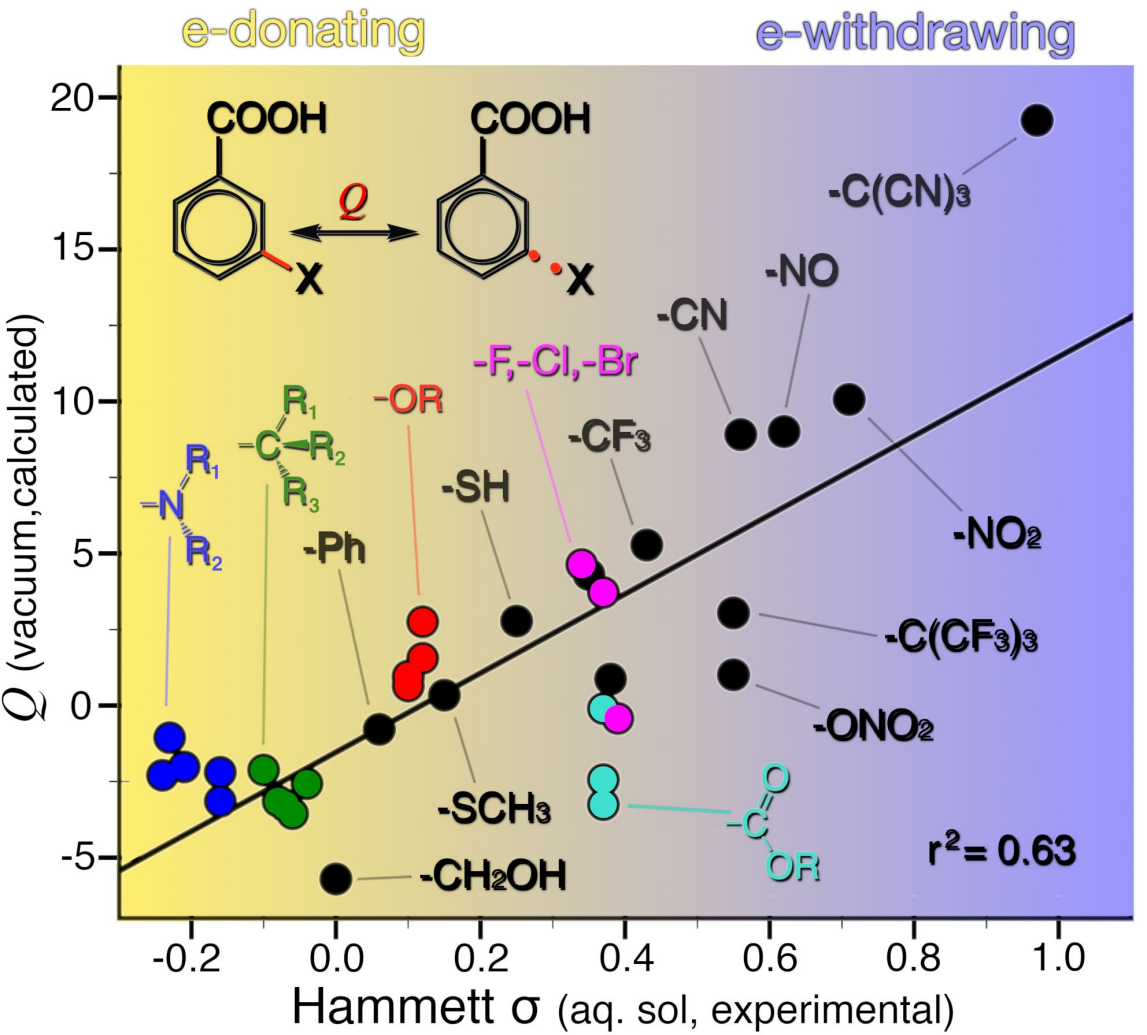
The *Q* descriptor calculated for the substituent bond plotted against the Hammett *σ* constant of meta‐substituted benzoic acids. Like earlier, colored circles indicate clustering of related chemical groups. A poorer correlation as compared to Figure 3 is ascribed to electronic effects that are more local to the substituent bond.

Next, we consider the dissociation of the substituent group itself, *i. e*., bonds exemplified by Figure 1d. Figure 4 shows that, in this case, the correlation between σ and *Q* is significantly decreased relative to the comparison in Figure 3.

The main outliers in Figure 4 include carbonyls, nitriles, and nitro groups, all of which are known to generate significant electric fields due to their intrinsic dipoles.[^1^, ^35^] Such fields are examples of *local* effects that affect the immediate surrounding, such as the substituent bond, more than regions further away, such as the carboxyl group. Other outliers in Figure 4 are the isophthalic esters (shown in cyan in Figure 4), which are known to exhibit a different kind of local effect. The length of the ester alkyl chain is known to have no influence on the reactivity of an isophthalic ester, measured in terms of its σ constant.[^2^, ^3^]

However, granted that differences in ester hydrolysis rates can be partially attributed to steric effects, the trend in rates suggest a local effect of the alkyl chain length on the ester group.^[36]^

We have noted a sensitivity of *Q* to effects local to the investigated bond (*viz*. Figure 1d) and seen how, in the absence of local effects (*viz*. Figure 1c), *Q* can correlate with known reactivity trends. What other limits might exist for *Q* as a reactivity descriptor? Can the equivalent analysis also work in non‐conjugated systems? To find out, we next investigate if *Q* can distinguish between field and resonance substituent effects.

### 2.4 *Q* as a Reactivity Descriptor in Non‐Conjugated Systems

**Figure 5:**
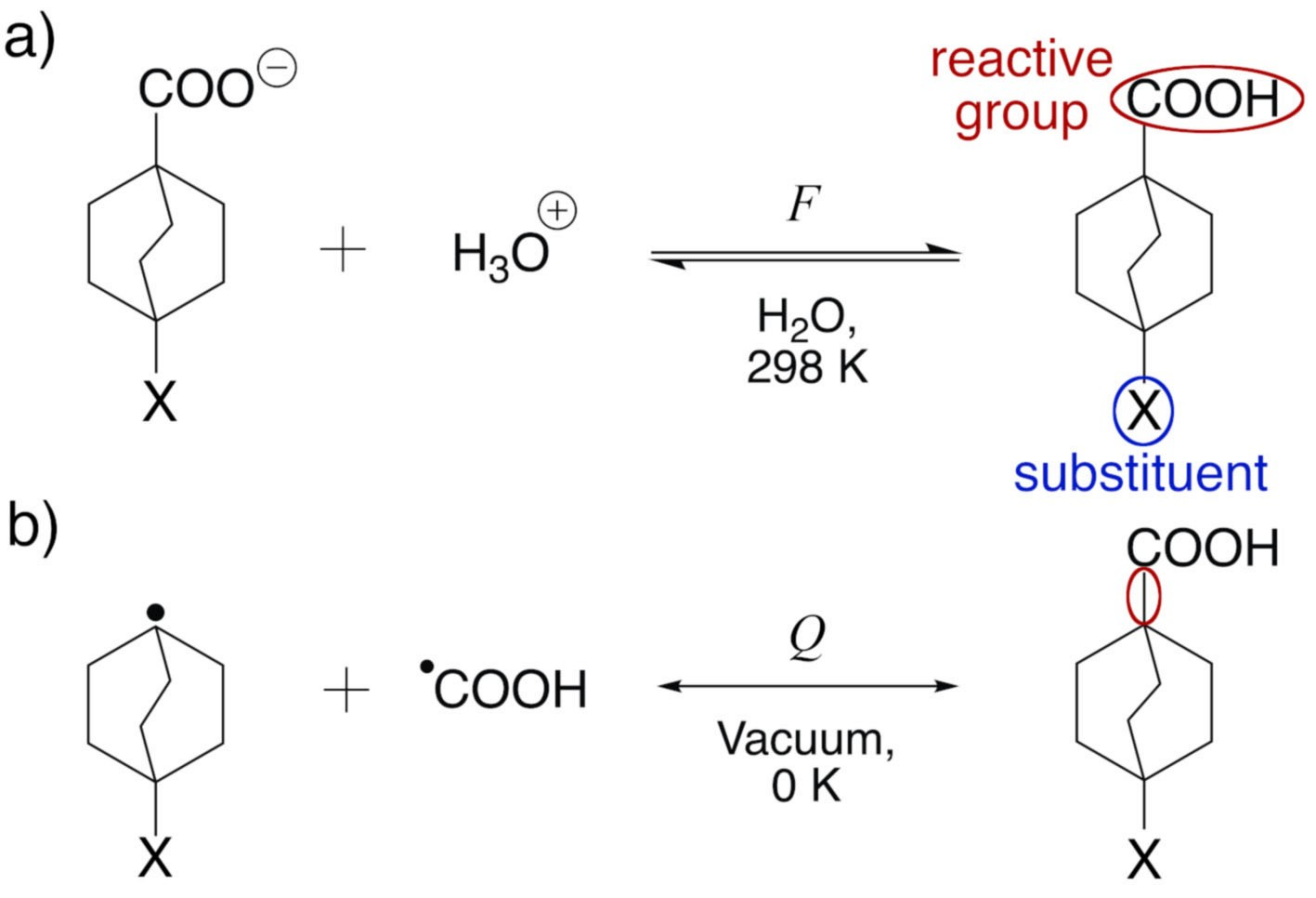
a) Field effects can be determined from changes in the p*K*_a_ of 4‐substituted bicyclo[2.2.2]octane carboxylic acids in aqueous solution. b) *Q* is here calculated for the bond between the carboxyl groups and the substituted bicyclo[2.2.2]octane fragment. Like before, the value of *Q* is independent of the direction of a chemical process and provides a value characteristic of the bonding process (Eq. 4).

To investigate if *Q* can be a reactivity descriptor also in non‐conjugated systems, we have calculated this metric for the carboxyl bond in 4‐substituted bicyclo[2.2.2]octane carboxylic acids (Figure 5b).

This class of compounds is conceptually non‐conjugated analogs of *para*‐substituted benzoic acids because the 4‐substituent and the carboxylic group have nearly identical distance and the same number of bonds in both types of compounds.^[37]^ These two families of compounds have been compared in the past to experimentally delineate the so‐called field (*F*) and resonance (*R*) effects of functional groups.^[3]^

Field effects typically refer to a combination of different through‐space effects. One kind of field effect is the electric field generated by the substituent, while another is through‐bond effects occurring due to polarization of σ bonds.[^1^, ^2^] In contrast, resonance effects are defined as those occurring only due to electron delocalization in conjugated π systems.^[1]^

The way experiments have been used to separate *F* from *R* is by first considering the overall effect of a substituent as a combination of *F* and *R* effects.^[38]^ This combination of effects is what is measured by σ in the traditional Hammett‐type experiments with, for example, benzoic acids (Figure 1a). By next considering non‐conjugated analogous of aromatic compounds, one can estimate the field effect of a chemical substituent separate from resonance effects. Figure 5a shows the acid‐base equilibrium for 4‐substituted bicyclo[2.2.2]octane carboxylic acids that have been used to derive tabulated field effects from measured changes in p*K*_a_ relative to a reference compound.[^3^, ^37^] Figure 5b shows our related approach for calculating *Q* for the carboxylic acid bond in these compounds.

**Figure 6:**
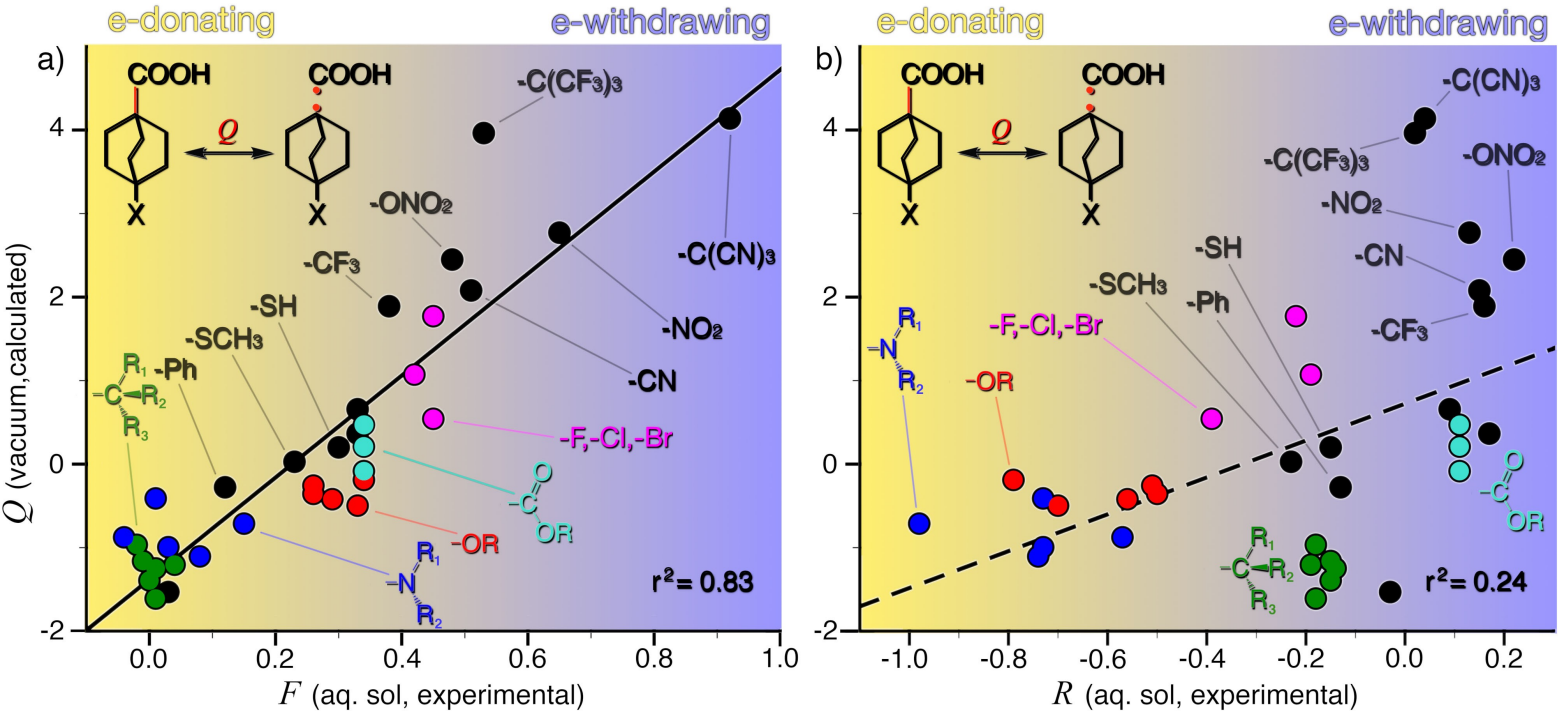
*Q* calculated for the carboxyl bonds in 4‐substituted bicyclo[2.2.2]octane carboxylic acids plotted against a) the field parameter F and b) the resonance parameter *R*.

Figure 6 compares the calculated *Q* values with experimental *F* and *R* parameters tabulated by Hansch *et al*.^[3]^ A linear regression shows a clear correlation between *Q* and *F (r^2^=0.83)*, a relationship that is largely mirrored when comparing *Q* and σ in conjugated systems (see Figures 2 and S1). In contrast, we find no clear correlation between *Q* and *R (r^2^=0.24)*.

A comparison of *Q* with *F* and *R* also for *meta*‐substituted benzoic acids can be found in the Supporting Information (Figure S2). *Q does* correlate with both *F (r^2^=0.78)* and *R (r^2^=0.49)* in *meta*‐substituted benzoic acids, although to a lower degree than with the σ constant (Figure 3). Having established, in our set of 35 substituents, a linear relationship between *Q* and *F*, and even more so between *Q* and σ, the question becomes: Can these relationships be used predictively?

### 2.5 Predicting Electronic Group Effects using *Q*

To test the predictive utility of *Q*, we have performed our analysis on an additional test set of 10 substituents. The models that we used to estimate σ and *F* as a function of *Q* are the linear regressions shown in Figure 3 and Figure 6a,

(5) $$\sigma_{\left(Q\right)}=0.14Q+0.28$$

and

(6) $$F_{\left(Q\right)}=0.14Q+0.24$$

where Eqs. (5) and (6) are used when the *Q* analysis is performed on benzoic acids and bicyclo[2.2.2]octane carboxylic acids, respectively. Because the test set is small it has not been chosen randomly, but instead designed to be clearly chemically distinct and outside the initial set used to derive Eqs. (5–6).

Experimental and predicted values of σ and *F* are shown in Table 1 for each subsituent in the test set. The root mean square error (RMSE) associated with the predicted data is ±0.12 and ±0.09 for σ and *F*, respectively. We note that the training set of 35 substituents provide a similar RMSE (±0.09 for both σ and *F*). Despite the inherent errors expected from the model, the *Q* analysis predicts the correct reactivity trends. This agreement opens up for qualitative prediction of subsituent groups outside of the 45 ones calculated.

**Table 1:** Hammett *σ* and *F* parameters predicted using Eqs. (5)–(6), *σ*_*(Q)*_, *F*_*(Q)*_, and experimental reference data, *σ_exp_*, *F_exp_*.^[a]^

Substituent	*σ*_***exp***_	*σ*_*(Q)*_	*F_exp_*	*F*_*(Q)*_
−CH_2_NH_2_	−0.03	0.13	0.04	0.01
−CH_2_Cl	0.11	0.26	0.13	0.26
−OCN	0.67	0.56	0.69	0.65
−NCO	0.27	0.27	0.31	0.44
−NC	0.48	0.44	0.47	0.51
−CONH_2_	0.28	0.16	0.26	0.28
−PH_2_	0.06	0.15	0.09	0.18
−SO_2_CH_3_	0.60	0.49	0.53	0.61
−SO_2_CF_3_	0.83	0.71	0.74	0.88
−N_3_	0.37	0.18	0.48	0.41
−N_5_	–	0.68	–	0.81

[a] This test set was selected to be chemically distinct from the data shown in Figures 3 and 6. The root mean square error associated with the predicted data is±0.12 and±0.09 for σ and *F*, respectively.

There exists a large number of substituents that are either too unstable or reactive to practically allow for experimental measurement of, for instance, σ or *F* values. One example is the highly energetic pentazolyl (−N_5_) group, which can be handled at low temperatures when present on suitably substituted arylpentazoles.^[39]^ The making of the kinetically more persistent pentazolate, *cyclo*‐N_5_^−^, anion, first in the gas phase,^[40]^ then in condensed phases^[41]^ have opened up for N_5_‐based reaction chemistry, and, possibly, for new kinds of pentazolyl‐substituted compounds. Our predicted σ and *F* values for the pentazolyl substituent indicate a strong electron‐withdrawal ability (Table 1). These predictions are in line with the known increased stability of aryl pentazoles in the presence of electron‐donating substituents^[39]^ and indicate that the electronic effects of the N_5_ group resemble those of SO_2_CF_3_.

## 3 Conclusions

In this work, we have evaluated the ability of a quantum chemically derived bonding descriptor, *Q*, to capture substituent effects in large molecules. *Q*, which is straightforward and fast to calculate using standard DFT methods, is found to correlate strongly with experimentally derived field effect parameters and Hammett σ constants in a test set of 45 substituted benzoic acids and their non‐conjugated analogs, 4‐substituted bicyclo[2.2.2]octane carboxylic acids. The correlation between *Q* and σ and *F* in aromatic and non‐aromatic compounds, respectively, opens for the possibility of predicting substituent effects in a new way. In particular, *Q* can serve as a fast computational substitute for σ and *F* parameters in cases where the experimental determination of the latter is challenging. As an example, we have applied the developed *Q‐*based protocol to predict the unknown σ and *F* values for the highly energetic pentazolyl, *cyclo*‐N_5_, substituent (Table 1).

Our work constitutes the first validation against experimental data of a different kind of chemical bonding analysis, the EQC‐EDA scheme when applied to larger molecules. The use of experimental data and reactivity scales in benchmarking of electronic structure analysis tools is desirable but relatively seldom practiced in a systematic fashion.[^31^, ^42^] The functional groups investigated in this work are commonly used and span a wide range of properties and structures. We would like to encourage the inclusion of the same set of compounds and associated σ, *F*, and *R* parameters in the evaluation of other electronic structure analysis methods. The benefit of such cross‐comparison, and our aim, is the gradual build‐up of comprehensive benchmarks that can facilitate both the comparison and development of electronic structure analysis methods, making them even better suited to serve synthetic chemists and materials scientists.

## Computational Methodology

All experimental σ, *F*, and *R* parameters are from Ref. [3]. Calculations have been performed at the M06‐2X^[43]^/aug‐cc‐pVTZ^[44]^ level of theory using Gaussian 16, revision B.01.^[45]^ All structures were confirmed as true minima on the potential energy surface through vibrational analyses at the same level of theory. Our results are robust with respect to smaller basis sets. A comparison with the aug‐cc‐pVDZ basis set can be found for a selection of molecules in Figure S3. Detailed lists of data for all considered substituents are provided in Tables S1, S2, S3, and S4. A full EQC‐EDA analysis on the training set of substituents is provided in Tables S5 and S6. The *Q*‐analysis was performed using a Python script provided at www.rahmlab.com. The script relies on the cclib parsing library^[46]^ and can interpret output from several common quantum chemistry programs.

## Conflict of interest

The authors declare no conflict of interest.

## Supporting information

As a service to our authors and readers, this journal provides supporting information supplied by the authors. Such materials are peer reviewed and may be re‐organized for online delivery, but are not copy‐edited or typeset. Technical support issues arising from supporting information (other than missing files) should be addressed to the authors.

Supplementary
