# Supplementary material for: Experimental Quantum Chemistry: A Hammett‐inspired Fingerprinting of Substituent Effects
Source: Chemphyschem. 2021 Feb 22;22(6):569–76. doi: 10.1002/cphc.202001053 (PMC8049055; doi:10.1002/cphc.202001053)
Supplement: Supplementary file 1 — Supplementary [file CPHC-22-569-s001.pdf]

ChemPhysChem

Supporting Information

Experimental Quantum Chemistry: A Hammett-inspired Fingerprinting of Substituent Effects

Francesco Sessa, Martina Olsson+, Fredrik Söderberg+, Fang Wang, and Martin Rahm*

Table of Contents

***Q*-ANALYSIS OF THE CARBOXYLIC O–H BOND IN *META*-SUBSTITUTED BENZOIC ACIDS.** 2

***Q* AS A REACTIVITY DESCRIPTOR, CALCULATED FOR CARBOXYL BONDS IN SUBSTITUTED BENZOIC ACIDS** 3

***Q*-ANALYSIS OF SUBSTITUENT GROUP BONDS IN *META*-SUBSTITUTED BENZOIC ACIDS** 5

***Q*-ANALYSIS OF CARBOXYL GROUP BONDS IN SUBSTITUTED BICYCLO[2.2.2]OCTANE CARBOXYLIC ACIDS** 6

THE *Q* DESCRIPTOR: FIELD AND RESONANCE EFFECTS IN *META*-SUBSTITUTED BENZOIC ACIDS 7

BASIS SET DEPENDENCE OF THE *Q*-ANALYSIS 9

EQC-EDA DETAILS: *META*-SUBSTITUTED BENZOIC ACIDS 10

EQC-EDA DETAILS: 4-SUBSTITUTED BICYCLO[2.2.2]OCTANE CARBOXYLIC ACIDS 11

COMMENTS ON THE CONNECTION BETWEEN *Q*, $\Delta\chi$ AND THE $\Delta(V_{NN}–E_{EE})$-TERM IN THE CASE OF A CHEMICAL BOND FORMATION. 12

REFERENCES 13

Q-Analysis of the Carboxylic O–H Bond in *meta*-Substituted Benzoic Acids.

Table S1. Hammett **σ** constants and *Q* values calculated for carboxylic OH bonds (see Figure 1b) in *meta*-substituted benzoic acids.

Substituent	σ_m	*Q*
NMe_2	-0.16	-8.90
NEt_2	-0.23	-9.63
NHMe	-0.21	-9.47
NH_2	-0.16	-9.17
NHEt	-0.24	-9.15
O*i*Pr	0.10	-9.36
OH	0.12	-8.28
OMe	0.12	-8.48
O*n*Pr	0.10	-9.37
OEt	0.10	-9.08
t-Bu	-0.10	-9.22
Me	-0.07	-8.05
Bu	-0.08	-8.97
*i*Pr	-0.04	-8.83
Et	-0.07	-8.42
*n*Pr	-0.06	-8.13
Ph	0.06	-9.33
CH_2OH	0.00	-8.48
SMe	0.15	-8.27
F	0.34	-8.10
SH	0.25	-5.87
Cl	0.37	-8.54
Br	0.39	-10.01
CHO	0.35	-8.24
COOH	0.37	-8.97
COOMe	0.37	-9.07
COOEt	0.37	-9.36
Ac	0.38	-8.84
CF_3	0.43	-9.63
$C(CF_3)_3$	0.55	-13.6
CN	0.56	-8.15
ONO_2	0.55	-9.38
NO_2	0.71	-8.60
NO	0.62	-8.53
$C(CN)_3$	0.97	-9.61

Q as a Reactivity Descriptor, Calculated for Carboxyl Bonds in Substituted Benzoic Acids

Figure S1 shows the comparison between the *Q* descriptor*,* calculated for the formation of the carboxyl bond in a set of 35 *para*-substituted benzoic acids, and the Hammett σ constant. The complete list of the substituents, along with detailed data of the analysis, can be found in Table S2.

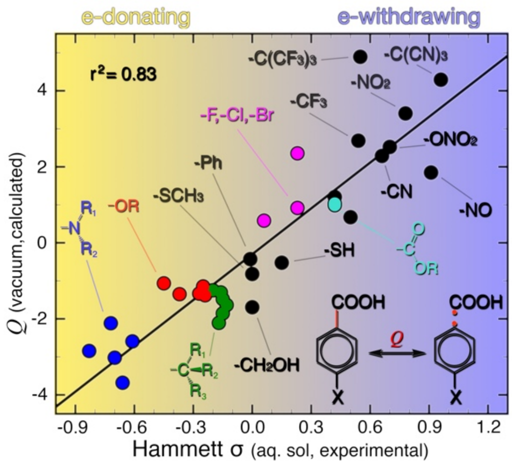

Figure S1. The *Q* descriptor calculated for the indicated carboxyl bond plotted against the Hammett σ constant of *para*-substituted benzoic acids. Colored circles indicate clustering of related chemical groups.

Table S2. Hammett **σ** constant and *Q* values for the formation of carboxyl bonds (see also Figure 1c) in *meta*- (σ_m, Q_m) and *para*- (σ_p, Q_p) substituted benzoic acids.

Substituent	σ_m	Q_m	σ_p	Q_p
NMe_2	-0.16	-3.29	-0.83	-2.85
NEt_2	-0.23	-3.37	-0.72	-2.12
NHMe	-0.21	-2.74	-0.70	-3.02
NH_2	-0.16	-3.68	-0.66	-3.68
NHEt	-0.24	-2.49	-0.61	-2.59
O*i*Pr	0.10	-1.77	-0.45	-1.07
OH	0.12	-1.05	-0.37	-1.35
OMe	0.12	-1.70	-0.27	-1.34
O*n*Pr	0.10	-1.62	-0.25	-1.15
OEt	0.10	-1.88	-0.24	-1.38
t-Bu	-0.10	-2.14	-0.20	-1.25
Me	-0.07	-2.05	-0.17	-2.11
Bu	-0.08	-2.01	-0.16	-1.30
*i*Pr	-0.04	-1.99	-0.15	-1.48
Et	-0.07	-2.16	-0.15	-1.86
*n*Pr	-0.06	-2.54	-0.13	-1.64
Ph	0.06	-0.77	-0.01	-0.43
CH_2OH	0.00	-2.56	0.00	-1.70
SMe	0.15	-2.06	0.00	-0.82
F	0.34	0.13	0.06	0.58
SH	0.25	-0.50	0.15	-0.52
Cl	0.37	0.44	0.23	0.92
Br	0.39	1.29	0.23	2.35
CHO	0.35	1.26	0.42	1.20
COOH	0.37	0.12	0.42	1.02
COOMe	0.37	-0.04	0.42	0.99
COOEt	0.37	-0.02	0.42	1.03
Ac	0.38	0.81	0.50	0.67
CF_3	0.43	1.32	0.54	2.69
$C(CF_3)_3$	0.55	3.98	0.55	4.89
CN	0.56	1.77	0.66	2.29
ONO_2	0.55	1.90	0.70	2.52
NO_2	0.71	2.61	0.78	3.40
NO	0.62	1.38	0.91	1.85
$C(CN)_3$	0.97	3.78	0.96	4.29

Q-Analysis of Substituent Group Bonds in *meta*-Substituted Benzoic Acids

Table S3. Hammett **σ** constants and *Q* values calculated for substituent bonds (see Figure 1d) in *meta*-substituted benzoic acids.

Substituent	σ_m	*Q*
NMe_2	-0.16	-2.20
NEt_2	-0.23	-1.06
NHMe	-0.21	-2.02
NH_2	-0.16	-3.15
NHEt	-0.24	-2.30
O*i*Pr	0.10	0.89
OH	0.12	2.75
OMe	0.12	1.55
O*n*Pr	0.10	0.64
OEt	0.10	0.97
t-Bu	-0.10	-2.12
Me	-0.07	-3.31
Bu	-0.08	-3.15
*i*Pr	-0.04	-2.57
Et	-0.07	-3.27
*n*Pr	-0.06	-3.56
Ph	0.06	-0.78
CH_2OH	0.00	-5.70
SMe	0.15	0.34
F	0.34	4.64
SH	0.25	2.77
Cl	0.37	-3.72
Br	0.39	-0.43
CHO	0.35	4.31
COOH	0.37	-0.10
COOMe	0.37	-2.44
COOEt	0.37	-3.26
Ac	0.38	0.87
CF_3	0.43	5.28
$C(CF_3)_3$	0.55	3.05
CN	0.56	8.91
ONO_2	0.55	1.01
NO_2	0.71	10.07
NO	0.62	9.01
$C(CN)_3$	0.97	19.25

Q-Analysis of Carboxyl Group Bonds in Substituted Bicyclo[2.2.2]octane Carboxylic Acids

Table S4. Field (*F*) and resonance (*R*) parameters and *Q* values of carboxyl bonds (see also Figure 4b) in 4-substituted bicyclo[2.2.2]octane carboxylic acids.

Substituent	*F*	*R*	*Q*
NMe_2	0.15	-0.98	-0.71
NEt_2	0.01	-0.73	-0.41
NHMe	0.03	-0.73	-0.99
NH_2	0.08	-0.74	-1.11
NHEt	-0.04	-0.57	-0.88
O*i*Pr	0.34	-0.79	-0.19
OH	0.33	-0.70	-0.50
OMe	0.29	-0.56	-0.42
O*n*Pr	0.26	-0.51	-0.26
OEt	0.26	-0.50	-0.36
t-Bu	-0.02	-0.18	-0.97
Me	0.01	-0.18	-1.61
Bu	-0.01	-0.15	-1.16
*i*Pr	0.04	-0.19	-1.20
Et	0.00	-0.15	-1.39
*n*Pr	0.01	-0.14	-1.25
Ph	0.12	-0.13	-0.28
CH_2OH	0.03	-0.03	-1.53
SMe	0.23	-0.23	0.03
F	0.45	-0.39	0.54
SH	0.30	-0.15	0.20
Cl	0.42	-0.19	1.07
Br	0.45	-0.22	1.77
CHO	0.33	0.09	0.66
COOH	0.34	0.11	0.47
COOMe	0.34	0.11	-0.08
COOEt	0.34	0.11	0.21
Ac	0.33	0.17	0.37
CF_3	0.38	0.16	1.89
$C(CF_3)_3$	0.53	0.02	3.96
CN	0.51	0.15	2.08
ONO_2	0.48	0.22	2.45
NO_2	0.65	0.13	2.77
NO	0.49	0.42	1.28
$C(CN)_3$	0.92	0.04	4.14

The *Q* Descriptor: Field and Resonance Effects in *meta*-Substituted Benzoic Acids

Figure S2 shows how the *Q* descriptor relates to field (*F*) and resonance (*R*) effects when the former is calculated for the carboxyl bond in the *meta*-substituted benzoic acids. The complete list of the substituents, along with detailed data of the analysis, can be found in Table S4. The correlation, measured as the coefficient of determination r^2, is significantly weaker (r^2 = 0.79 and 0.49 for *F* and *R*, respectively), compared to the relationship between *Q* and the Hammett σ parameter (*c.f.* r^2 = 0.90, Figure 2).

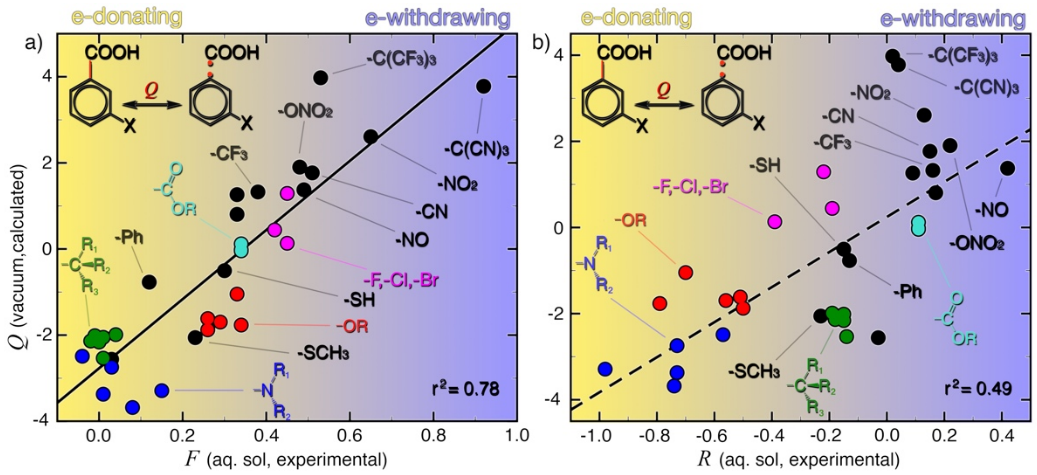

Figure S2. The *Q* descriptor calculated for the indicated carboxyl bond plotted against a) the field parameter F and b) the resonance parameter R. Colored circles indicate families of related chemical groups.

Table S5. Experimental field (*F*) and resonance (*R*) parameters together with computed *Q* values for the formation of carboxyl bonds in meta-substituted benzoic acids.

Substituent	*F*	*R*	*Q*
NMe_2	0.15	-0.98	-3.29
NEt_2	0.01	-0.73	-3.37
NHMe	0.03	-0.73	-2.74
NH_2	0.08	-0.74	-3.68
NHEt	-0.04	-0.57	-2.49
O*i*Pr	0.34	-0.79	-1.77
OH	0.33	-0.70	-1.05
OMe	0.29	-0.56	-1.70
O*n*Pr	0.26	-0.51	-1.62
OEt	0.26	-0.50	-1.88
t-Bu	-0.02	-0.18	-2.14
Me	0.01	-0.18	-2.05
Bu	-0.01	-0.15	-2.01
*i*Pr	0.04	-0.19	-1.99
Et	0.00	-0.15	-2.16
*n*Pr	0.01	-0.14	-2.54
Ph	0.12	-0.13	-0.77
CH2OH	0.03	-0.03	-2.56
SMe	0.23	-0.23	-2.06
F	0.45	-0.39	0.13
SH	0.30	-0.15	-0.50
Cl	0.42	-0.19	0.44
Br	0.45	-0.22	1.29
CHO	0.33	0.09	1.26
COOH	0.34	0.11	0.12
COOMe	0.34	0.11	-0.04
COOEt	0.34	0.11	-0.02
Ac	0.33	0.17	0.81
CF_3	0.38	0.16	1.33
$C(CF_3)_3$	0.53	0.02	3.98
CN	0.51	0.15	1.77
ONO_2	0.48	0.22	1.90
NO_2	0.65	0.13	2.61
NO	0.49	0.42	1.38
$C(CN)_3$	0.92	0.04	3.78

Basis Set Dependence of the *Q*-Analysis

To test for basis set dependence, the *Q*-analysis was performed on the initial set of 35 meta-substituted benzoic acids using both the M06-2X[1]/aug-cc-pVTZ[2] and M06-2X[1]/aug-cc-pVDZ[3] levels of theory. The two sets of computations are compared in Figure S3, which illustrates a minimal basis set dependence.

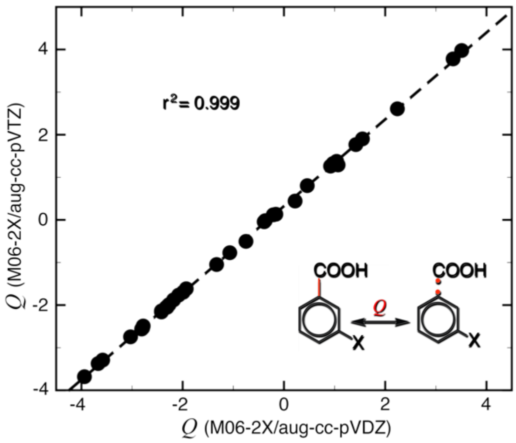

Figure S3. *Comparison of the* Q *descriptor calculated for the indicated carboxyl bond using the aug-cc-pVDZ and the aug-cc-pVTZ basis sets.*

EQC-EDA Details: *meta*-Substituted Benzoic Acids

Table S6. EQC-EDA terms for the formation of the carboxyl bond (see Figure 1b) in the investigated meta-substituted benzoic acids. All values are in eV.

Substituent	n	ΔE	$n\Delta\bar{\chi}$	$\Delta(V_{NN} - E_{ee})$	ΔV_{NN}	ΔE_{ee}
NMe_2	88	-4.84	5.54	-10.39	5366.47	5376.86
NEt_2	104	-4.86	5.76	-10.62	6185.07	6195.68
NHMe	80	-4.83	4.21	-9.04	4885.36	4894.40
NH_2	72	-4.99	6.69	-11.68	4434.78	4446.46
NHEt	88	-4.83	3.60	-8.42	5219.74	5228.17
O*i*Pr	96	-4.83	1.85	-6.69	5610.89	5617.58
OH	72	-4.84	0.11	-4.95	4516.72	4521.68
OMe	80	-4.83	1.68	-6.51	4904.48	4910.99
O*n*Pr	96	-4.83	1.49	-6.32	5629.62	5635.94
OEt	88	-4.83	2.12	-6.95	5264.65	5271.60
t-Bu	96	-4.86	2.76	-7.62	5775.32	5782.94
Me	72	-4.85	2.55	-7.39	4486.35	4493.74
Bu	96	-4.84	2.45	-7.30	5655.45	5662.74
*i*Pr	88	-4.85	2.40	-7.24	5274.51	5281.75
Et	80	-4.85	2.81	-7.66	4899.06	4906.72
*n*Pr	88	-4.85	3.72	-8.57	5311.11	5319.68
Ph	104	-4.84	-0.56	-4.29	5996.04	6000.33
CH_2OH	80	-4.84	3.78	-8.61	4981.26	4989.87
SMe	88	-4.76	2.52	-7.28	5340.47	5347.74
F	72	-4.84	-2.74	-2.10	4568.54	4570.64
SH	80	-4.83	-1.20	-3.63	4938.62	4942.26
Cl	80	-4.84	-3.49	-1.35	5023.58	5024.93
Br	98	-4.83	-5.54	0.70	6049.95	6049.25
CHO	78	-4.84	-5.48	0.64	4783.37	4782.74
COOH	86	-4.84	-2.71	-2.13	5260.02	5262.15
COOMe	94	-4.84	-2.31	-2.52	5595.78	5598.30
COOEt	102	-4.83	-2.38	-2.46	5894.86	5897.32
Ac	86	-4.85	-4.38	-0.47	5256.84	5257.31
CF_3	96	-4.83	-5.62	0.78	5820.01	5819.23
$C(CF_3)_3$	168	-4.83	-12.02	7.19	9502.19	9495.00
CN	76	-4.81	-6.66	1.85	4680.27	4678.42
ONO_2	94	-4.83	-7.01	2.18	5674.35	5672.18
NO_2	86	-4.82	-8.70	3.88	5300.47	5296.59
NO	78	-4.84	-5.75	0.91	4794.40	4793.49
$C(CN)_3$	108	-4.81	-11.49	6.68	6394.72	6388.04

EQC-EDA Details: 4-Substituted Bicyclo[2.2.2]octane Carboxylic Acids

Table S7. EQC-EDA terms for the formation of the carboxyl bond (see Figure 4b) in the investigated 4-substituted bicyclo[2.2.2]octane carboxylic acids. All values are in eV.

Substituent	n	ΔE	$n\Delta\bar{\chi}$	$\Delta(V_{NN}-E_{ee})$	ΔV_{NN}	ΔE_{ee}
NMe_2	108	-4.41	-0.64	-3.77	6978.14	6981.91
NEt_2	124	-4.41	-1.30	-3.11	7616.38	7619.49
NHMe	100	-4.40	-0.01	-4.39	6598.76	6603.15
NH_2	92	-4.39	0.23	-4.63	6226.36	6230.98
NHEt	108	-4.40	-0.27	-4.13	6906.06	6910.19
O*i*Pr	116	-4.40	-1.78	-2.62	7274.17	7276.79
OH	92	-4.39	-1.10	-3.29	6235.35	6238.64
OMe	100	-4.40	-1.28	-3.13	6611.59	6614.72
O*n*Pr	116	-4.40	-1.64	-2.77	7200.78	7203.55
OEt	108	-4.40	-1.42	-2.98	6921.90	6924.88
t-Bu	116	-4.41	-0.08	-4.33	7333.45	7337.78
Me	92	-4.39	1.34	-5.73	6210.29	6216.02
Bu	116	-4.40	0.35	-4.75	7163.13	7167.88
*i*Pr	108	-4.40	0.45	-4.85	6956.50	6961.35
Et	100	-4.40	0.86	-5.26	6585.28	6590.54
*n*Pr	108	-4.40	0.55	-4.94	6887.91	6892.85
Ph	124	-4.39	-1.59	-2.81	7529.71	7532.51
CH_2OH	100	-4.40	1.17	-5.56	6602.29	6607.86
SMe	108	-4.39	-2.26	-2.13	6978.80	6980.93
F	92	-4.39	-3.39	-1.00	6245.19	6246.19
SH	100	-4.38	-2.63	-1.75	6613.84	6615.59
Cl	100	-4.38	-4.54	0.16	6618.48	6618.32
Br	118	-4.38	-6.06	1.69	7502.15	7500.47
CHO	98	-4.38	-3.63	-0.75	6501.95	6502.70
COOH	106	-4.39	-3.23	-1.16	6887.68	6888.84
COOMe	114	-4.36	-2.00	-2.36	7176.49	7178.86
COOEt	122	-4.39	-2.66	-1.73	7494.26	7496.00
Ac	106	-4.39	-3.00	-1.39	6876.16	6877.56
CF_3	116	-4.39	-6.34	1.95	7390.98	7389.02
$C(CF_3)_3$	188	-4.39	-10.90	6.51	10601.22	10594.71
CN	96	-4.37	-6.73	2.36	6393.28	6390.92
ONO_2	114	-4.39	-7.57	3.18	7245.38	7242.21
NO_2	106	-4.38	-8.27	3.88	6913.77	6909.89
NO	98	-4.39	-5.01	0.62	6523.22	6522.61
$C(CN)_3$	128	-4.37	-11.22	6.85	7872.43	7865.58

Comments on the Connection Between Q, $\Delta\bar{\chi}$ and the $\Delta(V_{NN}-E_{ee})$-Term in the Case of a Chemical Bond Formation.

We refer to Ref. [4] for an in-depth discussion of the physical meaning of different values of Q. Below, we go through a brief overview and begin by reminding how we partition the bond energy,[5]

$$\Delta E = n\Delta\bar{\chi} + \Delta V_{NN} - \Delta E_{ee}, \qquad \text{(S1)}$$

and how Q is defined,[4]

$$Q = \left(n\Delta\bar{\chi} - \Delta(V_{NN} - E_{ee})\right)/\Delta E. \qquad \text{(S2)}$$

where n is the number of electrons, ΔE is the change in total energy over a transformation, $\Delta\bar{\chi}$ is the change in the average electron energy and $\Delta(V_{NN} - E_{ee})$ is the change in the balance of the electrostatic repulsion between equally charged particles, nuclei, and electrons, respectively.

In what follows, we look at two hypothetical reference situations that can but need not occur, in which a bond is formed and either of two options, a or b, is true:

a. $\Delta E = n\Delta\bar{\chi}$
b. $\Delta E=\Delta(V_{NN}-E_{ee})$

In both cases, we assume that the system is stabilized upon bond formation, i.e., $\Delta E<0$. What do the two situations correspond to?

a. The $\Delta(V_{NN} - E_{ee}) = 0$ Reference in Chemical Bonding

In the first reference scenario, the total bond energy is described by the $n\Delta\bar{\chi}$-term, i.e., we have $\Delta E = n\Delta\bar{\chi}$ and $\Delta(V_{NN}-E_{ee})=0$ in Eq. S1. The $\bar{\chi}$-term quantifies the average electron binding energy, but can also be conceptually and approximately interpreted as the average energy of the occupied molecular orbitals. In other words, in our study, the $n\Delta\bar{\chi}$-term quantifies the overall orbital stabilization when a bond is formed. This example coincides with simple (e.g. Hückel) Molecular Orbital theory picture of a chemical bond, in which the stabilization of molecular orbitals is the sole reason for a bond's formation. The Q-value for such a bond is equal to 1.00.

The other term of the energy expression, $\Delta(V_{NN}-E_{ee})$, which is equal to zero in this example, quantifies how well changes to electron repulsions over a transformation are compensated by change to the nuclear repulsion. In situations where the bond formation is driven by the $n\Delta\bar{\chi}$-term being negative, but where the $\Delta(V_{NN}-E_{ee})$-term is non-zero, Q will instead take a value larger than zero.

In this work, we have seen that a positive Q-value is computed for carboxyl bonds in *meta*-substituted benzoic acid when the substituent is electron-withdrawing. This result means that, for substituents with a stronger electron-withdrawing ability, the bonding of the carboxyl group is dominated by orbital stabilization, the $n\Delta\bar{\chi}$-term. We can rationalize this correspondence by recalling that electron-withdrawing groups have a known destabilizing effect on the molecular orbitals of the benzyl radical,[6] which then become stabilized upon bond formation.

b. The $\Delta\bar{\chi} = 0$ Reference in Chemical Bonding

In our second reference example, the total bond energy is instead described by the $\Delta(V_{NN}-E_{ee})$-term, i.e. $\Delta E=\Delta(V_{NN}-E_{ee})$ and $n\Delta\bar{\chi}=0$. In this case, which corresponds to $Q = -1.00$, electron rearrangement goes beyond just compensating for the nuclear repulsion, and instead enables the formation of the bond.

The $n\Delta\bar{\chi}=0$ example can be perceived as uncomfortable from a MO theory perspective. In this situation, a bond is formed without a net orbital stabilization. Note, however, that the $\Delta\bar{\chi}$-term quantifies the average change in the energies of occupied orbitals, and that individual orbitals, such as the HOMO, can still be stabilized. Nevertheless, processes driven by $\Delta(V_{NN}-E_{ee})$ might correspond to situations where conventional MO- and electronegativity-based arguments are misleading.[4,5] The Q values for such bonds are always less than zero, $Q < 0$.

We note that the full EQC-EDA analysis (Table S5 and S6) shows that while the combined electrostatic $\Delta(V_{NN}-E_{ee})$-term contains information related to reactivity, the individual ΔV_{NN} and ΔE_{ee}-terms show no significant correlation with σ.

References

[1] Y. Zhao, D. G. Truhlar, *Theor. Chem. Acc.* **2008**, *120*, 215.
[2] R. A. Kendall, T. H. Dunning, Jr., R. J. Harrison, *J. Chem. Phys.* **1992**, *96*, 6796.
[3] A. K. Wilson, D. E. Woon, K. A. Peterson, T. H. Dunning, Jr., *J. Chem. Phys.* **1999**, *110*, 7667.
[4] M. Rahm, R. Hoffmann, *J. Am. Chem. Soc.* **2016**, *138*, 3731–3744.
[5] M. Rahm, R. Hoffmann, *J. Am. Chem. Soc.* **2015**, *137*, 10282.
[6] T. H. Fisher, S. M. Dershem, M. L. Prewitt, *J. Org. Chem.* **1990**, *55*, 1040.
